# Breastmilk or infant formula? Content analysis of infant feeding advice on breastmilk substitute manufacturer websites

**DOI:** 10.1017/S1368980021003451

**Published:** 2023-05

**Authors:** Jennifer L Pomeranz, Xiangying Chu, Oana Groza, Madeline Cohodes, Jennifer L Harris

**Affiliations:** 1 School of Global Public Health, New York University, New York, NY, USA; 2 UConn Rudd Center for Food Policy and Obesity, University of Connecticut, Hartford, CT, USA

**Keywords:** infant health, infant formula, breast-feeding/breastmilk, BMS marketing

## Abstract

**Objective:**

To evaluate messages about infant feeding on breastmilk substitute (BMS) manufacturer websites directed at US caregivers and compare information and portrayals of breast-feeding/breastmilk with that of infant formula (IF) feeding.

**Design:**

We conducted a content analysis of US BMS companies’ websites. A codebook was created through an iterative process to identify messages and images about breast-feeding/breastmilk and IF feeding, including benefits or issues associated with each, and direct-to-consumer marketing practices that could discourage breast-feeding.

**Setting:**

Data were collected in 2019–2020 and analysed in 2020–2021 for US websites of five IF manufacturers.

**Participants:**

The websites of Similac, Enfamil and Gerber, which collectively represent approximately 98 % of the US IF market, and two US organic brands, Earth’s Best and Happy Baby.

**Results:**

Websites contained more messages about breast-feeding/breastmilk than IF but were significantly more likely to mention benefits to baby of IF (44 %) than breast-feeding/breastmilk (<26 %), including significantly more statements that IF provides brain, neural and gastrointestinal benefits; 40 % of breast-feeding/breastmilk content was dedicated to breast-feeding problems (e.g. sore nipples). Twice as many screenshots compared IF brands favourably to breastmilk than as superior to other brands. Certain companies displayed images indicating ease of IF feeding and difficulty of breast-feeding.

**Conclusions:**

Substantial messaging on BMS manufacturer websites encouraged IF feeding and discouraged breast-feeding. Health professionals should discourage their patients from visiting these websites and the US government should regulate misleading claims. Companies should refrain from providing breast-feeding advice and align their US marketing with the International Code of Marketing of Breast-milk Substitutes.

## Introduction

Optimal nutrition during a baby’s first year of life is critical for future health outcomes^(1,2)^. In 2011, the US Surgeon General issued a Call to Action to Support Breastfeeding, in which she recommended that infant formula (IF) should be marketed in a way that does not discourage breast-feeding in the USA^(1)^. The Call to Action suggested evaluating marketing claims to identify practices that may negatively influence breast-feeding decisions and further proposed that IF marketers should be held accountable for complying with the International Code of Marketing of Breast-milk Substitutes (the Code)^(1)^. The World Health Assembly adopted the Code in 1981 to provide recommendations to countries and breastmilk substitute (BMS) manufacturers on methods to promote and protect breast-feeding, which included prohibiting direct-to-consumer promotion of BMS^(2)^.

Although the WHO identified a key priority for member countries to eliminate the advertisement and promotion of BMS to the general public, the USA is one of few countries that have not adopted any portion of the Code into law^(3)^. As a result, marketing and labelling techniques that are prohibited in other countries are regularly used to sell IF in the USA^(3)^. One such technique includes BMS companies maintaining websites with substantial educational materials and other resources for parents about feeding their baby in their first year^(4)^.

The WHO recommends exclusive breast-feeding until 6 months of age and continued breast-feeding through 2 years of age^(5)^. The American Academy of Pediatrics recommends exclusive breast-feeding for the first 6 months of life and continued breast-feeding until 1 year of age if mutually beneficial to mother and baby^(6)^. However, in the USA, 19·2 % of breastfed infants born in 2017 were supplemented with IF before 2 d of age^(7)^, and less than half (46·9 %) of infants in 2015 were exclusively breastfed by 3 months^(8)^. IF sales in the USA were $2·65 billion in 2018^(9)^ and prior to the COVID-19 pandemic, sales were projected to reach $4·450 billion in 2020^(10)^.

Many factors influence the decision to breastfeed or use IF including breast-feeding support, career and work schedules, cultural beliefs, and the availability of information, but advertising and promotion of IF plays a critical role^(11,12)^. It is important for public health practitioners, nutritionists and healthcare providers working on infant health and nutrition to understand that the marketing messages parents are receiving directly from BMS manufacturers, especially on websites targeting new parents^(13)^.

Previous research found BMS manufacturer marketing influences social norms and attitudes around infant feeding^(11)^. In particular, BMS advertising has been found to idealise the use of BMS^(14)^, for example, by using unsubstantiated health, nutrition and breastmilk comparison claims that lead caregivers to believe that IF is ‘as good as’ or even ‘better’ than breastmilk^(4,11,15–17)^. At the same time, these advertisements diminish maternal confidence in breast-feeding and the benefits of breastmilk^(11)^. BMS manufacturers have been found to position themselves as experts in public health and infant feeding^(18)^. One method they accomplish this is by aligning their companies with ‘the public health establishment’^(19)^ and maintaining company websites with advice on infant feeding^(4)^. These websites have been found to include subtle messaging to encourage IF feeding, ‘reduce guilt about not breast-feeding’ and discourage breast-feeding by positioning breast-feeders as a ‘minority’ group with ‘one set of beliefs’^(4)^.

Research to date has not compared messaging that discusses breast-feeding/breastmilk *v*. IF feeding on BMS manufacturer websites directed to US consumers. The current study examines how major IF companies communicate with the public about the benefits and challenges of breast-feeding and IF feeding on their US websites. Through a content analysis of BMS manufacturer webpages, we examined messages, through text and image that were dedicated to infant feeding and compared information provided about the benefits and difficulties of breast-feeding/breastmilk with IF feeding. Additionally, we evaluated techniques used to support IF feeding through direct-to-consumer marketing practices, such as product discounts and providing access to infant feeding support representatives.

## Methods

Websites from five IF manufacturers were chosen for evaluation. The manufacturers included the three most popular brands of IF in the USA: Similac, Enfamil and Gerber, which collectively represent approximately 98 % of the IF market share^(20)^, and two US organic brands, Earth’s Best and Happy Baby, that have a history of advertising their IF online^(4)^.

### Procedures and measures

For each of the five websites, researchers identified all webpages related to feeding an infant with breastmilk or IF through text, image or both. Researchers also identified additional content that included messages and marketing practices that could discourage breast-feeding as noted in the Code^(2)^ and/or the Surgeon General’s recommendations related to marketing of IF^(1)^. Specifically, the Code recommends against the use of information or educational materials, marketing, text (e.g. terms such as ‘humanised’ to describe IF), or images that promote or idealise bottles, bottle-feeding, or IF, or that discourage breast-feeding^(2)^. The Code suggests prohibiting the advertisement or other forms of promotion to the general public of IF, including direct-to-consumer marketing practices to reduce the price of formula through coupons or discounts^(2)^. Additionally, manufacturers and distributors are instructed not to provide access to ‘professional service representatives’ that mimic healthcare professionals^(2)^.

Data were collected in two phases: August–September 2019 and September 2019–January 2020. One research assistant who was blind to the purpose of the study first accessed every webpage of each website and took screenshots of all relevant material. Relevant content included all messaging (including text and images) about feeding infants 0–12 months, IF, breastmilk, breast-feeding, supplementing, pumping and direct-to-consumer IF marketing practices in the form of basic website material, ‘pop-ups’ from the manufacturer, articles, guides, parent reviews/testimonials and academic resources. All webpages requiring additional clicks to review the full material were expanded. Excluded content included webpages providing directions on IF preparation, and those exclusively focused on the mother’s health, diet or pregnancy, the sale of products without additional text, and complementary food or non-IF BMS (e.g. toddler milks) feeding and products. A total of 545 screenshots were captured during this first data collection. To ensure that all relevant content was captured, a second researcher who was not blind to the purpose of the study used the same methodology and captured relevant screenshots not originally identified. A total of 287 additional screenshots were added through this second data collection. Each screenshot was the size of an average computer screen, so each webpage could result in more than one screenshot. As some manufacture websites contained substantially more content per webpage, this method allowed researchers to standardise the amount of content between websites.

A codebook was created through an iterative process to identify three types of content: 1) messages about breast-feeding/breastmilk, including benefits or issues associated with breast-feeding/breastmilk; 2) messages about IF feeding, including benefits or issues associated with IF feeding and 3) additional related marketing including: images that support or discourage IF feeding or breast-feeding (e.g. by suggesting the ease of formula feeding or difficulty of breast-feeding); direct-to-consumer sales incentives (e.g. product discounts, coupons, and rewards programmes); and providing access to healthcare professionals or other infant feeding support representatives. The codebook is included as an Appendix.

The principal investigator created the initial version of the codebook and trained two research assistants to code the screenshots. Screenshots were coded to indicate the presence of each message in the codebook; each screenshot could contain more than one message. After coding one BMS manufacturer’s website chosen at random, the researchers discussed inconsistencies and amended the codebook as necessary. The two coders recoded the same website and achieved greater than 90 % agreement. Both coders then coded two additional websites and each coder coded one additional website, with random quality checks by the principal investigator. After assessing inter-coder agreement, the principal investigator determined that five questions contained the majority of disagreements and should be removed from the study. The coders evaluated any remaining disagreements and then recoded the screenshots with disagreements.

### Statistical analysis

Descriptive statistics (frequency and percentage of screenshots) were calculated for all codes within the three content areas in total and for each BMS manufacturer. The ratio of number and percent of screenshots that mentioned IF feeding *v*. breast-feeding/breastmilk in total and by manufacturer were calculated. Comparisons between the proportion of screenshots that indicated specific benefits of breast-feeding/breastmilk *v*. IF were conducted using chi-squared tests and Fisher’s exact tests (for small cell counts when the assumptions of the chi-squared test were violated). We also calculated the frequency of screenshots that mentioned specific issues with breast-feeding/breastmilk by manufacturer and percentage of all screenshots regarding breast-feeding/breastmilk, and the ratio of screenshots that mentioned breast-feeding issues *v*. positive mentions of breast-feeding. All analyses were conducted using R software (Version 4·0·3). Statistical significance was assessed at *P* < 0·05.

## Results

In total, 678 relevant screenshots across the 5 websites were included in the study, ranging from 77 screenshots for Earth’s Best to 255 for Enfamil (see Table 1). Of this total, 303 screenshots were related to breast-feeding/breastmilk and 263 screenshots were related to IF feeding; 127 screenshots mentioned both. An additional 239 screenshots contained additional messages that encouraged IF use, including promotions, access to health professionals and images. Happy Baby stood out as having almost 4·5 times the number of screenshots dedicated to breast-feeding/breastmilk (*n* 130) as compared to IF feeding (*n* 29). Gerber also mentioned breast-feeding more often than IF. In contrast, Enfamil and Similac mentioned IF more often than breast-feeding/breastmilk.

**Table 1:** Content of BMS website screenshots related to breastmilk/breast-feeding, infant formula feeding and additional messages that may discourage breast-feeding

			Company*									
	Total (*n* 678)		Enfamil (*n* 241)		Happy Baby (*n* 212)		Similac (*n* 96)		Gerber (*n* 95)		Earth’s Best (*n* 34)	
Content of screenshots	# of screenshots	% of total	# of screenshots	% of company total	# of screenshots	% of company total	# of screenshots	% of company total	# of screenshots	% of company total	# of screenshots	% of company total
Total screenshots with mentions of breast-feeding/breastmilk	303	45	76	32	130	61	24	25	53	56	20	59
Total screenshots with mentions of infant formula feeding	263	39	123	51	29	14	44	46	40	42	27	79
Ratio of screenshots that mentioned breast-feeding *v*. infant formula feeding=	1·15		0·62		4·48		0·55		1·33		0·74	
*Claims promoting brand as superior*												
– Brand is mentioned as superior, better, preferred, #1, #1 recommended by experts	15	2	1	0	4	2	0	0	1	1	9	26
– Infant formula or brand discussed as closest to breastmilk	33	5	17	7	5	2	6	6	3	3	2	6
*Additional messages that may discourage breast-feeding*												
Images that support infant formula feeding or suggest ease of formula feeding or difficulty of breast-feeding												
– Bottle and/or nipple not being used for feeding	32	5	9	4	17	8	2	2	4	4	0	0
– Baby feeding with both hands on the bottle	21	3	14	6	6	3	1	1	0	0	0	0
– Mother breast-feeding with one hand on her breast	28	4	0	0	25	12	3	3	0	0	0	0
– Mother breast-feeding with both hands on her breast	9	1	0	0	9	4	0	0	0	0	0	0
Direct-to-consumer promotions												
– Coupons, discounts, rewards	196	29	100	41	40	19	47	49	7	7	2	6
– Providing contact information for, or access to a health professional or consultant	335	49	81	34	193	91	46	48	9	9	6	18

* N = number of screenshots that mentioned breastmilk/breast-feeding, infant formula feeding and/or other relevant content on each company’s website. Screenshots may contain more than one type of content and multiple types of messages so numbers add up to more than 100 %.

Of the total, 2 % of screenshots compared their brand to other BMS brands with terms such as ‘superior’, ‘better’, ‘preferred’, ‘#1’ or ‘#1 recommended by experts’, while 5 % compared IF or their brand to breastmilk using terms such as ‘closest to’, ‘most similar’, ‘inspired by’ or ingredients/nutrients ‘found in’ breastmilk. More than 50 % of comparisons to breastmilk appeared on the Enfamil website.

Manufacturers’ websites also contained images that support IF feeding or suggest ease of formula feeding and/or difficulty of breast-feeding, including bottles or nipples not being used for feeding (5 % of screenshots), an infant with two hands on the bottle while feeding (3 % of screenshots, two-thirds by Enfamil), a mother breast-feeding with her own hand on her breast (4 % of screenshots, primarily by Happy Baby) and a mother breast-feeding with her own two hands on her breast (1 %, all Happy Baby). Only Earth’s Best had none of these images.

Furthermore, 29 % of screenshots displayed or mentioned a coupon, discount or reward related to IF; more than 50 % of these were on the Enfamil website. Almost one-half of screenshots (49 %) provided contact information for, or access to an infant feeding support representative, health professional, or other consultant; Happy Baby provided this information more than any other company and it appeared on 91 % of Happy Baby’s total screenshots.

As shown in Table 2, screenshots that mentioned IF feeding on BMS manufacturer websites were significantly more likely to mention benefits for baby (44 %) compared to screenshots that mentioned the benefits of breast-feeding/breastmilk for baby (26 %). In addition, the types of benefits mentioned differed for breast-feeding/breastmilk as opposed to IF. There were significantly more statements that breastmilk promotes bonding/skin-to-skin contact and immunity than similar statements for IF. Conversely, websites made significantly more statements that IF provides the benefits of DHA and arachidonic, brain, neural and eye health, as well as reducing stomach and gastrointestinal issues, than similar statements about breastmilk. Additionally, organic, non-GM organisms and natural were commonly used to describe IF, but not breastmilk.

**Table 2:** Comparison between screenshots that mentioned specific benefits of breast-feeding/breastmilk *v*. infant formula (n and % of total values)

Total screenshots that mentioned benefits for baby*	Breast-feeding/breastmilk		Infant formula feeding		*P*-value
	*n* 78		*n* 116		
	*n*	% of total**	*n*	% of total**	
*Specific benefits mentioned*					
– Vitamins, minerals, nutrients, healthy, health, nutritious	49	62·8	79	68·1	0·54
– DHA, ARA, brain, neural, eye	17	21·8	52	44·8	0·001
– Reduce stomach, gastrointestinal issues, probiotics	11	14·1	33	28·4	0·02
– Immunity, reduce likelihood of sickness	26	33·3	17	14·7	0·03
– Organic, non-GMO, natural	0	0·0	19	16·4	<0·001
– Bonding; skin-on-skin	6	7·7	1	0·9	0·02
– Reduce skin issues, rash, eczema, acne	3	3·8	2	1·7	0·39
Total screenshots mentioning benefits (% of total screenshots that mentioned breast-feeding/breastmilk or infant formula feeding)	78	25·7	116	44·1	<0·001

DHA, docosahexaenoic acid; ARA, arachidonic acid; GMO, genetically modified organism.

* Total screenshots includes all screenshots that mentioned benefits of breastmilk/breast-feeding or infant formula feeding. Each screenshot may contain more than one benefits, so numbers add to more than 100 %.

** N’s = 303 (# of screenshots that mentioned breast-feeding) and 263 (# of screenshots that mentioned infant formula feeding).

Shading indicates a significantly higher percentage (*P* < 0·05).

Finally, BMS manufacturer websites often mentioned issues related to breast-feeding, which appeared on 40 % of screenshots that mentioned breast-feeding (see Table 3). The most common issues mentioned were breastmilk supply (e.g. rapid, slow) on 20 % of screenshots about breast-feeding, and infant latching (19 %); followed by mentions of nipples (e.g. sore, chapped) (11 %), clogged ducts and engorgement (6 % each), and leaking breastmilk (4 %). Happy Baby was responsible for the majority of these screenshots (61 % of the total). In addition, Happy Baby, Similac and Gerber mentioned breast-feeding issues on 30 % or more of their screenshots that mentioned breast-feeding (57 %, 42 % and 30 %, respectively). In contrast, Earth’s Best mentioned breast-feeding issues on just 10 % of breast-feeding screenshots.

**Table 3:** Content of screenshots that mentioned breast-feeding/breastmilk by manufacturer

			Company*									
	Total (*n* 303)		Earth’s Best (*n* 20)		Enfamil (*n* 76)		Gerber (*n* 53)		Happy Baby (*n* 130)		Similac (*n* 24)	
	Total	% of total	Total	% of company total	Total	% of company total	Total	% of company total	Total	% of company total	Total	% of company total
# of screenshots that mentioned issues with breast-feeding (% of total screenshots with breast-feeding mentions)	122	40	2	10	20	26	16	30	74	57	10	42
*Breast-feeding issues mentioned*												
– Breastmilk supply (e.g. rapid, slow, reduced, neutral, not enough milk, good)	61	20	2	10	13	17	10	19	31	24	5	21
– Latch or latching	57	19	0	0	11	14	5	9	35	27	6	25
– Leaking breastmilk mentioned	12	4	0	0	3	4	1	2	7	5	1	4
– Engorgement	18	6	0	0	3	4	5	9	8	6	2	8
– Clogged ducts	19	6	0	0	2	3	2	4	14	11	1	4
– Nipples mentioned (e.g. sore, chapped, infection, neutral, other)	33	11	0	0	5	7	9	17	16	12	3	13
# of screenshots with positive breast-feeding mentions (% of total screenshots with breast-feeding mentions)	78	26	14	70	17	22	17	32	23	18	7	29
Ratio of # of screenshots with breast-feeding issues *v*. positive breast-feeding mentions	1·56		0·14		1·18		0·94		3·22		1·43	

* N = total screenshots that mentioned breast-feeding/breastmilk. Each screenshot may mention more than one issue, so numbers may add up to more than 100 %.

## Discussion

This is the first study to examine and compare information and portrayals of breast-feeding/breastmilk with IF feeding on BMS manufacturer websites directed at US consumers. This research identified substantial messaging that encouraged IF feeding and discouraged breast-feeding in ways that contradict the US Surgeon General’s recommendations and violate the Code. Marketing practices directed towards US consumers that would be legally suspect in other countries included promoting IF and bottles directly to the public, using price reductions and coupons to induce sale, idealising IF in text and images, providing contact information for professional service representatives, claiming health and nutritional benefits of IF over breastmilk, and using terms to ‘humanise’ IF^(2,21)^.

In addition to overt messages that were contrary to the Code, BMS websites engaged in subtle messaging to encourage IF feeding or discourage breast-feeding/breastmilk. Most notably, this study identified unrealistic images suggesting the relative ease of bottle-feeding, with infants seeming to hold their own bottle, as compared to the ‘work’ involved with breast-feeding, with women holding their breasts. Further, even seemingly innocuous practices, such as providing information to contact a health professional, has been documented as a marketing strategy to increase BMS sales. Previous research found that once a woman calls a BMS telephone advice line, ‘there is a significant correlation with her ultimately buying’ that company’s BMS^(19)^.

Perhaps most concerning was the current study’s corroboration of previous research finding that BMS marketing expressly discouraged breast-feeding^(11)^. IF manufacturer websites more frequently discussed breast-feeding/breastmilk than IF, and portrayals of breast-feeding frequently suggested potential problems a mother may experience. In contrast, the vast majority of screenshots dedicated to IF described the benefits of IF. Even if the breast-feeding messages were framed in a way to suggest solutions to the issues identified, the repeated communication about problems such as reduced breastmilk supply or sore nipples, coupled with images of women holding their breasts to breastfeed, implies that breast-feeding is difficult, painful work, thereby discouraging breast-feeding. BMS manufacturers should refrain from providing information or advice on breast-feeding in all of their marketing materials.

BMS manufacturer websites also contained more than twice as many screenshots comparing their brand favourably to breastmilk than as superior to other brands even though company websites are ostensibly designed to promote their brand over other brands. Rather, BMS manufacturer websites focused more on promoting the IF category or their brand as a choice in contrast to breast-feeding and breastmilk as opposed to another brand^(22)^. Such messaging may also discourage breast-feeding.

It is noteworthy that the two companies with the most screenshots related to infant feeding, Enfamil and Happy Baby, appeared to use opposite marketing strategies. Enfamil’s text and images focused on IF feeding, while Happy Baby’s text and images focused on breast-feeding. Yet, both companies position themselves as experts on child and infant health and nutrition. Notably, Happy Baby had a non-moving link at the bottom of every screen with an option to ‘Chat with our Nutritionists & Lactation Specialists!’ which is why almost every screenshot from their website provided contact to a professional service representative.

The USA has not adopted policies to implement the Code likely in part because of the structure of the US marketplace^(23)^. The US government historically supports the interests of US-based corporations over public health, including on the world stage – even opposing World Health Assembly resolutions^(23)^. Moreover, the US government, through the US Department of Agriculture (USDA), is the country’s largest purchaser of IF through the federal Special Supplemental Program for Women, Infants and Children (WIC), which provides IF to participants.

Another reason why the USA may not adopt policies to implement the Code is because the First Amendment of the US Constitution protects commercial speech, which is speech that proposes a commercial transaction^(24)^. Many of the advertising and labelling practices discouraged by the Code are considered protected commercial speech in the USA. Nonetheless, the Code and the US Surgeon General recommended that independent of government regulation to implement the Code, BMS manufacturers should align their marketing practices according to the principles and aims of the Code^(2)^. IF manufacturers that sell products in the USA have not abided by these recommendations.

Due to the absence of BMS manufacturer self-regulation, it is important to identify avenues for future regulatory action. Although truthful commercial speech is protected by the First Amendment, marketing that is false, deceptive or unfair is not similarly protected^(24)^. The US Federal Trade Commission (FTC) and state attorneys general have consumer protection authorities that include addressing false, deceptive and unfair marketing practices on company websites. The FTC and state attorneys general should use this authority to address the issues identified in this study including the use of unsubstantiated structure/function claims, marketing messages that ‘humanise’ IF and those that portray IF as more protective of infant health than breastmilk. These claims should be considered false, unfair or inherently misleading and thus subject to regulation or restriction^(24)^.

Because BMS websites display products, the regulation of IF labelling is also relevant. The US Food and Drug Administration (FDA) has authority over IF labels but lacks the same authority as the FTC to address unsubstantiated structure/function claims on food labels. The FDA previously proposed draft guidance for IF labelling that suggested it is a good practice for structure/function claims to be evidence-based^(25)^; however, the guidance did not apply to claims comparing IF to breastmilk and the FDA never finalised the document.

Congress should provide the FDA with the authority to regulate structure function claims, at least for BMS, including restricting unfair breastmilk comparison claims on product labels. The FDA should then regulate these claims. In addition, the USDA could use its purchasing power for WIC to only purchase IF products from companies that refrain from engaging in practices contrary to the Surgeon General’s recommendations and the Code.

Like all studies, this study has limitations. We may not have captured all relevant screenshots, although our method of using two researchers, with one blind to the study design, decreased the likelihood that there would be substantial oversight. Our study’s evaluation was restricted to BMS companies’ websites in English directed at US consumers, so we did not review specialty websites, websites in other languages or those with information directed at medical professionals^(26–28)^. These websites are ripe for evaluation. It is also noteworthy that BMS companies frequently change their website content, so the screenshots we captured may represent content that no longer exists. The codebook was developed to identify types of messages that previous research indicates may discourage breast-feeding. However, additional research is needed to assess the ever-evolving marketing messages used to sell IF; the extent consumers, and especially new parents, read and rely on these websites; caregivers’ interpretation of specific messages; and the potential impact of these websites on their infant feeding decisions.

In conclusion, BMS company websites appear to target pregnant women and new mothers with IF marketing disguised as feeding advice and support. The US government should actively regulate marketing messages on websites and product labelling, including addressing false, unfair, and deceptive claims and statements. In the meantime, public health practitioners, nutritionists and healthcare providers play a major role in providing information to support the nutritional health of infants. Since BMS company websites may contradict their advice and undermine public health recommendations, health professionals should counsel their patients to avoid these websites as a source of information. BMS manufacturers should also immediately refrain from providing breast-feeding advice, ensure that all claims are evidence-based, truthful and not misleading and align their marketing directed at US consumers with the recommendations of the US Surgeon General and the Code.

## Supplementary material

For supplementary material accompanying this paper visit https://doi.org/10.1017/S1368980021003451. click here to view supplementary material
