# Supplementary material for: Breastmilk or infant formula? Content analysis of infant feeding advice on breastmilk substitute manufacturer websites
Source: Public Health Nutr. 2021 Sep 14;26(5):934–42. doi: 10.1017/S1368980021003451 (PMC10346044; doi:10.1017/S1368980021003451)
Supplement: Supplementary file 1 [file S1368980021003451sup001.pdf]

Appendix. Infant formula company website coding

Start of Block: Default Question Block

Q1 Formula company

- ○ Earth's Best (1)
- ○ Enfamil (2)
- ○ Gerber (3)
- ○ Happy Tot (4)
- ○ Similac (5)

Q2 PDF page # (e.g., 1 or 23 or 123)

____

Q3 Does a health/medical organization, doctor, or other healthcare provider suggest, support, or recommend Breastfeeding/Breastmilk; did a health/medical org or provider write the content or is a medical study referenced on this page RELATED TO BREASTFEEDING OR BREASTMILK?

- ○ Yes (1)
- ○ No (2)

Q4 Does a health/medical organization, doctor, or other healthcare provider suggest, support, or recommend infant formula or this brand; did a health/medical org or provider write the content or is a medical study referenced on this page RELATED TO INFANT FORMULA OR the BRAND? (includes statement #1 recommended by pediatricians)

○ Yes (1)

○ No (3)

Q5 Does this page discuss breastfeeding or breastmilk (BF/BM)?

○ Yes (1)

○ No (2)

Display This Question:

If Does this page discuss breastfeeding or breastmilk (BF/BM)? = Yes

Q6 Is BF/BM discussed as superior, better, or preferred as directly compared to IF?

○ Yes (1)

○ No (2)

Display This Question:

If Does this page discuss breastfeeding or breastmilk (BF/BM)? = Yes

Q7 Is BF/BM mentioned as positive for mother or caretaker?

○ Yes (1)

○ No (2)

Display This Question:

If Is BF/BM mentioned as positive for mother or caretaker? = Yes

Q8 How BF/BM positive for mother or caretaker?

- ☐ bonding; skin-on-skin (1)
- ☐ healthy for mother (e.g., stress relief) (2)
- ☐ convenient (3)
- ☐ other (6)

Display This Question:

If Does this page discuss breastfeeding or breastmilk (BF/BM)? = Yes

Q9 Is BF/BM mentioned as having nutrients or otherwise positive for baby?

- ○ Yes (1)
- ○ No (2)

Display This Question:

If Is BF/BM mentioned as having nutrients or otherwise positive for baby? = Yes

Q10 How BF/BM positive for baby?

- ☐ vitamins, minerals, nutrients, healthy, health, nutritious (253)
- ☐ DHA, ARA, brain, neural, eye (254)
- ☐ immunity, reduces likelihood of sickness (256)
- ☐ skin, rash, eczema, acne (257)
- ☐ stomach, GI reasons, probiotics (258)
- ☐ bonding; skin-on-skin (259)
- ☐ organic, non-GMO, natural (261)

Display This Question:

If Does this page discuss breastfeeding or breastmilk (BF/BM)? = Yes

Q11 Is breast milk supply mentioned (e.g., rapid, slow, reduced, neutral, not enough milk, good)?

◯ Yes (1)

◯ No (2)

Display This Question:

If Does this page discuss breastfeeding or breastmilk (BF/BM)? = Yes

Q12 Is latch or latching mentioned?

◯ Yes (1)

◯ No (2)

Display This Question:

If Does this page discuss breastfeeding or breastmilk (BF/BM)? = Yes

Q13 Is leaking (nipples/breasts/milk) mentioned?

◯ Yes (1)

◯ No (2)

Display This Question:

If Does this page discuss breastfeeding or breastmilk (BF/BM)? = Yes

Q14 Is engorgement mentioned?

◯ Yes (1)

◯ No (2)

Display This Question:

If Does this page discuss breastfeeding or breastmilk (BF/BM)? = Yes

Q15 Are clogged ducts mentioned?

- ○ Yes (1)
- ○ No (2)

Display This Question:

If Does this page discuss breastfeeding or breastmilk (BF/BM)? = Yes

Q16 Are nipples mentioned (e.g., sore, chapped, infection, neutral, other)?

- ○ Yes (1)
- ○ No (2)

Display This Question:

If Does this page discuss breastfeeding or breastmilk (BF/BM)? = Yes

Q17 Is baby's spitting up mentioned in relation to BF/BM?

- ○ Yes (1)
- ○ No (2)

Display This Question:

If Does this page discuss breastfeeding or breastmilk (BF/BM)? = Yes

Q18 Is baby's crying/fussiness mentioned in relation to BF/BM?

- ○ Yes (1)
- ○ No (2)

Display This Question:

If Does this page discuss breastfeeding or breastmilk (BF/BM)? = Yes

Q19 Is baby's gas mentioned in relation to BF/BM?

- ◯ Yes (1)
- ◯ No (2)

Display This Question:

If Does this page discuss breastfeeding or breastmilk (BF/BM)? = Yes

Q20 Is baby's skin (e.g., rash, eczema, itching) mentioned in relation to BF/BM?

- ◯ Yes (1)
- ◯ No (2)

Display This Question:

If Does this page discuss breastfeeding or breastmilk (BF/BM)? = Yes

Q21 Is baby's constipation mentioned in relation to BF/BM?

- ◯ Yes (1)
- ◯ No (2)

Display This Question:

If Does this page discuss breastfeeding or breastmilk (BF/BM)? = Yes

Q22 Is baby's diarrhea mentioned in relation to BF/BM?

- ◯ Yes (1)
- ◯ No (2)

Display This Question:

If Does this page discuss breastfeeding or breastmilk (BF/BM)? = Yes

Q23 Is baby's not gaining weight/failure to thrive/not growing mentioned in relation to BF/BM?

- ◯ Yes (1)
- ◯ No (2)

End of Block: Default Question Block

Start of Block: Block 1

Q24 Is the word pump or pumping mentioned?

- ◯ Yes (1)
- ◯ No (2)

Q25 Is supplementing mentioned. This = ongoing feeding of both BM and IF (neither exclusive or adding IF to BM) mentioned using this or other words?

- ◯ Yes (1)
- ◯ No (2)

Q26 Is transitioning from BF/BM to IF, switching from BF/BM to infant formula mentioned? This is discussing moving from BF/BM to IF.

- ◯ Yes (1)
- ◯ No (2)

End of Block: Block 1

Start of Block: Block 2

Q27 Does this page discuss infant formula (IF)?

○ Yes (1)

○ No (2)

Display This Question:

If Does this page discuss infant formula (IF)? = Yes

28 Is infant formula (IF) or Brand mentioned as superior, better, or preferred compared to BF/BM

○ Yes (1)

○ No (3)

Display This Question:

If Does this page discuss infant formula (IF)? = Yes

29 Is BRAND mentioned as superior, better, preferred, #1, #1 recommended by experts, to other brands?

○ Yes (1)

○ No (2)

Display This Question:

If Does this page discuss infant formula (IF)? = Yes

Q30 Is infant formula (IF) or Brand mentioned as closest to breastmilk? (e.g., "closest to" "most similar" "inspired by" , has ingredients/nutrients "found in" breastmilk, etc.)

○ Yes (1)

○ No (2)

Display This Question:

If Does this page discuss infant formula (IF)? = Yes

Q31 Is infant formula (IF) explicitly compared to food/nutrients from foods (e.g., blueberries, fish)?

- ○ Yes (1)
- ○ No (2)

Display This Question:

If Does this page discuss infant formula (IF)? = Yes

Q32 Is IF mentioned as positive for mother or caregiver?

- ○ Yes (1)
- ○ No (2)

Display This Question:

If Is IF mentioned as positive for mother or caregiver? = Yes

Q33 How is IF positive for mother or caregiver?

- ☐ bonding; skin-on-skin (1)
- ☐ healthy for mother (2)
- ☐ convenience (includes mother working) (3)
- ☐ allows others to feed (4)
- ☐ other (5)

Display This Question:

If Does this page discuss infant formula (IF)? = Yes

Q34 Is IF or BRAND mentioned as having nutrients or otherwise positive for baby?

○ Yes (1)

○ No (2)

Display This Question:

If Is IF or BRAND mentioned as having nutrients or otherwise positive for baby? = Yes

Q35 How is IF/Brand mentioned as having nutrients or otherwise positive for baby?

▢ vitamins, minerals, nutrients, nutritious, healthy, health (6)

▢ DHA, ARA, brain, neural, eye (7)

▢ immunity, reduce likelihood of sickness (9)

▢ skin, rash, eczema, acne (10)

▢ stomach, GI reasons, probiotics (11)

▢ bonding, skin-on-skin (12)

▢ organic, Non-GMO, natural (13)

Display This Question:

If Does this page discuss infant formula (IF)? = Yes

Q36 Is baby's gas mentioned in relation to IF?

○ Yes (1)

○ No (2)

Display This Question:

If Does this page discuss infant formula (IF)? = Yes

Q37 Is baby's skin (e.g., rash, eczema, itching) mentioned in relation to IF?

○ Yes (1)

○ No (2)

Display This Question:

If Does this page discuss infant formula (IF)? = Yes

Q38 Is baby's constipation mentioned in relation to IF?

○ Yes (1)

○ No (2)

Display This Question:

If Does this page discuss infant formula (IF)? = Yes

Q39 Is baby's diarrhea mentioned in relation to IF?

○ Yes (1)

○ No (2)

Display This Question:

If Does this page discuss infant formula (IF)? = Yes

Q40 Is baby's spitting up mentioned in relation to IF?

○ Yes (1)

○ No (2)

Display This Question:

If Does this page discuss infant formula (IF)? = Yes

Q41 Is baby's crying/fussiness mentioned in relation to IF?

◯ Yes (1)

◯ No (2)

Display This Question:

If Does this page discuss infant formula (IF)? = Yes

Q42 Is baby's not gaining weight/failure to thrive/not growing mentioned in relation to IF?

◯ Yes (1)

◯ No (2)

End of Block: Block 2

Start of Block: Block 3

Q43 Are there RELEVANT images? (bottle, nipple, feeding, pump/pumping) ONLY code WHAT YOU SEE after seeing full image.

◯ Yes (1)

◯ No (2)

Display This Question:

If Are there RELEVANT images? (bottle, nipple, feeding, pump/pumping) ONLY code WHAT YOU SEE after s... = Yes

Q44 How many RELEVANT images? (use numbers, e.g., 3) (bottle, nipple, feeding, pump/pumping) ONLY code WHAT YOU SEE after seeing full image.

____

Display This Question:

If Are there RELEVANT images? (bottle, nipple, feeding, pump/pumping) ONLY code WHAT YOU SEE after s... = Yes

Q45 Image: bottle/nipple without feeding (e.g., on counter, just held by adult)?

◯ Yes (1)

◯ No (2)

Display This Question:

If Are there RELEVANT images? (bottle, nipple, feeding, pump/pumping) ONLY code WHAT YOU SEE after s... = Yes

Q46 Image: pump/pumping?

◯ Yes (1)

◯ No (3)

Display This Question:

If Are there RELEVANT images? (bottle, nipple, feeding, pump/pumping) ONLY code WHAT YOU SEE after s... = Yes

Q47 Image: baby 1 hand on bottle?

◯ Yes (1)

◯ No (2)

Display This Question:

If Are there RELEVANT images? (bottle, nipple, feeding, pump/pumping) ONLY code WHAT YOU SEE after s... = Yes

Q48 Image: baby 2 hands on bottle?

◯ Yes (1)

◯ No (2)

Display This Question:

If Are there RELEVANT images? (bottle, nipple, feeding, pump/pumping) ONLY code WHAT YOU SEE after s... = Yes

Q49 Image: adult feeder(s) 1 hand on bottle?

- Yes (1)
- No (2)

Display This Question:

If Are there RELEVANT images? (bottle, nipple, feeding, pump/pumping) ONLY code WHAT YOU SEE after s... = Yes

Q50 Image: adult feeder(s) 2 hands on bottle?

- Yes (1)
- No (2)

Display This Question:

If Are there RELEVANT images? (bottle, nipple, feeding, pump/pumping) ONLY code WHAT YOU SEE after s... = Yes

Q51 Image: baby 1 hand on breast/mother's body?

- Yes (1)
- No (2)

Display This Question:

If Are there RELEVANT images? (bottle, nipple, feeding, pump/pumping) ONLY code WHAT YOU SEE after s... = Yes

Q52 Image: baby 2 hands on breast/mother's body?

- Yes (1)
- No (2)

Display This Question:

If Are there RELEVANT images? (bottle, nipple, feeding, pump/pumping) ONLY code WHAT YOU SEE after s... = Yes

Q53 Image: mom 1 hand on breast?

- Yes (1)
- No (2)

Display This Question:

If Are there RELEVANT images? (bottle, nipple, feeding, pump/pumping) ONLY code WHAT YOU SEE after s... = Yes

Q54 Image: mom 2 hands on breast?

- Yes (1)
- No (2)

End of Block: Block 3

Start of Block: Block 4

Q55 Are there coupons, discounts, rewards available/mentioned/shown?

- Yes (1)
- No (2)

Q56 Does this web-page provide contact information for, or access to a health professional/consultant?

- ○ Yes (1)
- ○ No (2)

---

*Q57 Does web-page explicitly suggest speaking to a lactation consultant or expert about BF/IF or decisions related to BF/IF (not just providing contact information/access to one)?

- ○ Yes (1)
- ○ No (3)

End of Block: Block 4

Start of Block: Block 5

Q58 Does web-page give advice about switching between formula types (e.g., gentle, lactose free etc.)?

- ○ Yes (1)
- ○ No (3)

---

*Q59 Does the web-page offer solutions to problems?

- ○ Yes (1)
- ○ No (2)

---

*Q60 Is infant formula or brand presented as solution to problems identified?

- ◯ Yes (1)
- ◯ No (3)

---

*Q61 Is breastfeeding/breastmilk presented as solution to problems identified?

- ◯ Yes (1)
- ◯ No (3)

---

*Q62 Does the web-page provide supportive or empowering statements about feeding decisions or emphasize choice?

- ◯ Yes (1)
- ◯ No (2)

Q63 Does page mention that water can be given before 6 months?

- ◯ Yes (1)
- ◯ No (3)

Q64 Is BF/BM discussed in relation to exclusive feeding for 0-6 months?

- ◯ Yes (1)
- ◯ No (3)

Q65 Is Infant Formula mentioned as exclusive for 0-6 months?

- ◯ Yes (1)
- ◯ No (3)

Q66 Additional Notes?

____

---

*Q67 Which coder are you?

End of Block: Block 5

---

*Questions excluded from results.
